# Supplementary material for: Hafnium—an optical hydrogen sensor spanning six orders in pressure
Source: Nat Commun. 2017 Jun 5;8:15718. doi: 10.1038/ncomms15718 (PMC5465374; doi:10.1038/ncomms15718)
Supplement: Supplementary Information — Supplementary Figures, Supplementary Tables, Supplementary Notes, Supplementary Methods and Supplementary References [file ncomms15718-s1.pdf]

Supplementary Figures

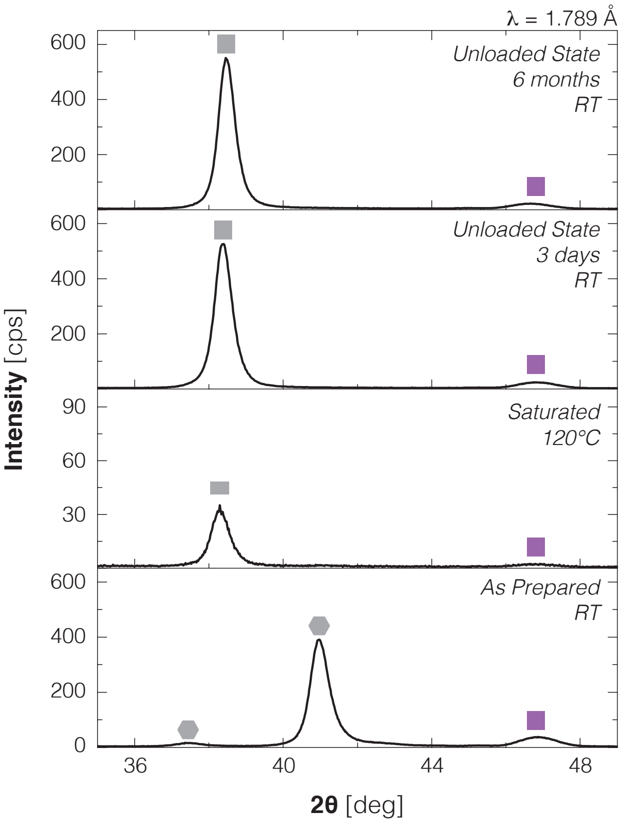

Supplementary Figure 1 | X-Ray Diffraction Patterns. X-ray diffraction patterns measured in-situ at different states of a Pd-capped HfH_x film (40 nm). The as prepared state is measured in air at room temperature. The saturated state is obtained by exposing the film to 10^{+3} Pa at 120°C until it reached a stable state (after six hours). The unloaded states are obtained by exposing the film (after saturation) to air for resp. three days and six months. The symbols ⬢, ■, ▬ (all HfH_x) and ■ (Pd) above each intensity peak indicates the corresponding structures, identified by means of the JCPDS-database (see Supplementary Table 2)[2]. The reduced intensity observed at the saturated state is due to the shielding of Argon gas within the cell.

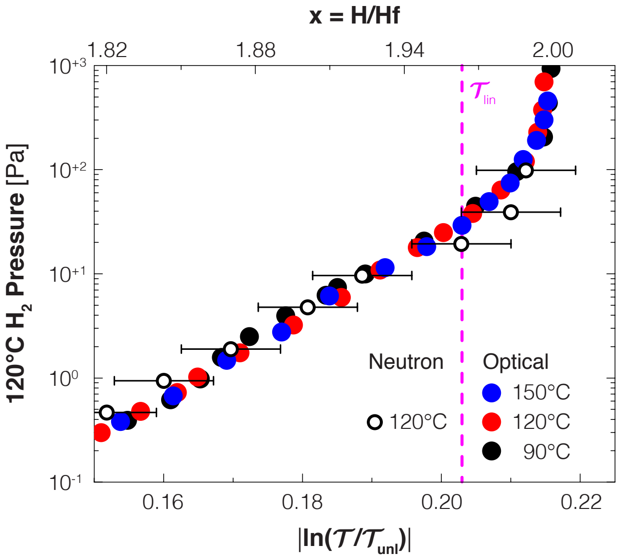

Supplementary Figure 2 | $\mathbf{HfH}_x$ Master Curve. Partial pressure-transmission-isotherms (P$\mathcal{T}$Is) normalized to the 120°C data. In addition, the relation between the hydrogen fraction x and the pressure P as obtained by neutron reflectometry at 120°C is shown. The magenta dashed line indicates the optical state $\mathcal{T}_{\mathrm{lin}}$, corresponding to a pressure of 30 Pa at 120°C. The error bars follows from the fitting procedure (see methods section main text).

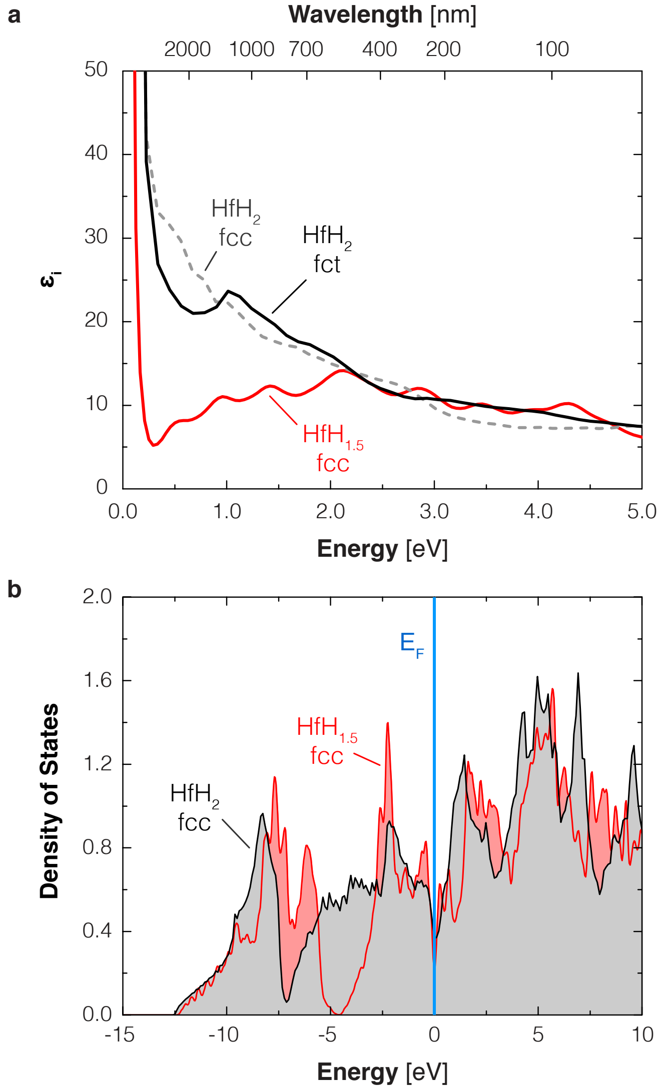

Supplementary Figure 3 | Calculated HfH$_x$ Dielectric Constants. **a** Imaginary part of the dielectric function ε comparing fcc and fct HfH_2 with fcc $HfH_{1.5}$. **b** Density of states plotted for fcc HfH_2 and $HfH_{1.5}$.

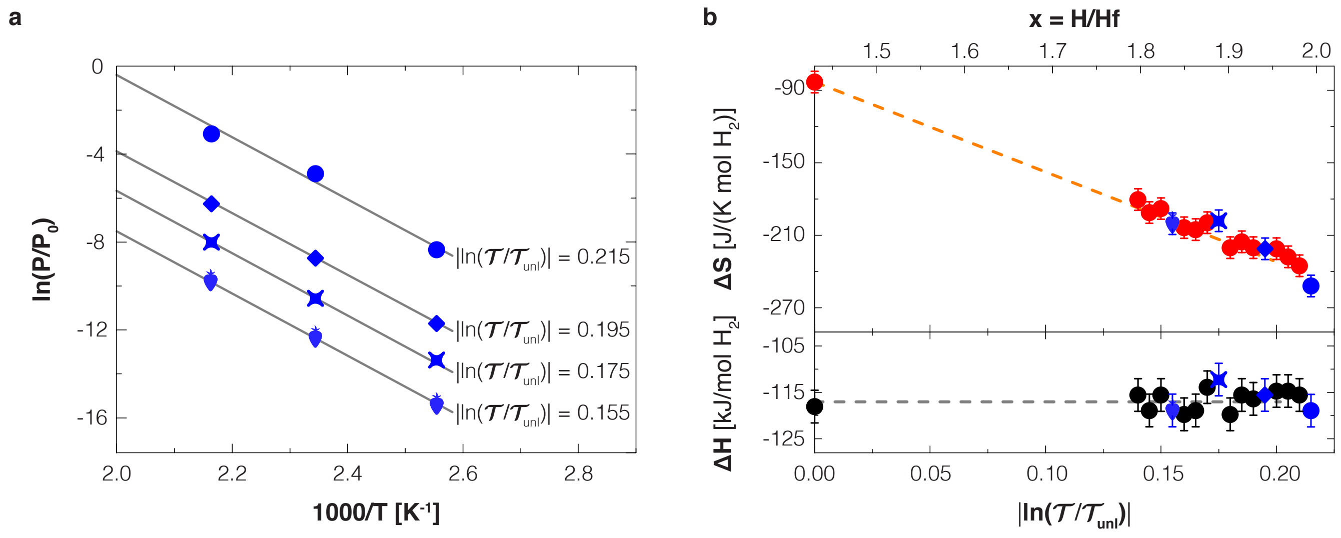

Supplementary Figure 4 | Thermodynamics of HfH$_x$. a The construction of the Van 't Hoff plot for four different hydrogen fractions corresponding to four selected values for the optical transmission. The constant slope of the curves reflects the constant enthalpy of dissolution. **b** Plot of the entropy ΔS and enthalpy ΔH as a function of the optical transmission $\ln(\mathcal{T}/\mathcal{T}_{\mathrm{unl}})$, where we used Fig. 3 to relate $\ln(\mathcal{T}/\mathcal{T}_{\mathrm{unl}})$ to the hydrogen fraction x as in Fig. 5. The blue symbols are the values obtained from the corresponding Van 't Hoff analysis in **a**. The entropy and enthalpy values extrapolated to $\ln(\mathcal{T}/\mathcal{T}_{\mathrm{unl}}) = 0$ correspond well to the values derived by Mintz[1] at the hcp/fcc phase boundary.

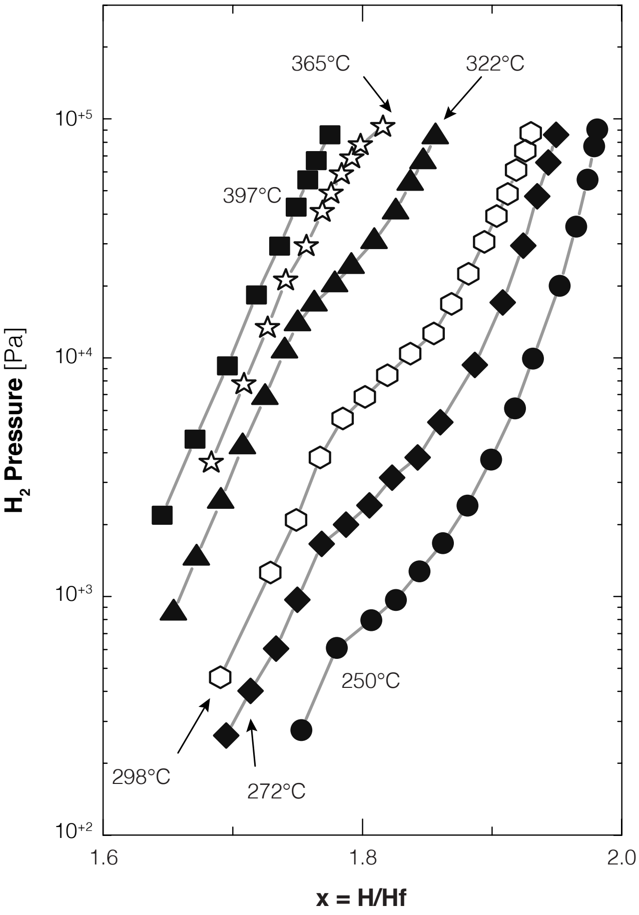

Supplementary Figure 5 | Pressure-Composition-Isotherms of Bulk HfH_x. Partial pressure-composition-isotherms of bulk HfH_x at various temperatures between 250 – 400°C, replotted from Mintz[1] by converting the hydrogen concentration $c = x/(1 + x)$ in to the hydrogen fraction x and the hydrogen pressure P from mmHg to Pa. We observe straight lines in the fcc phase, which is followed by a more curved behavior. The kink at $x \approx 1.78$ has been attributed to the transition to the fct phase.

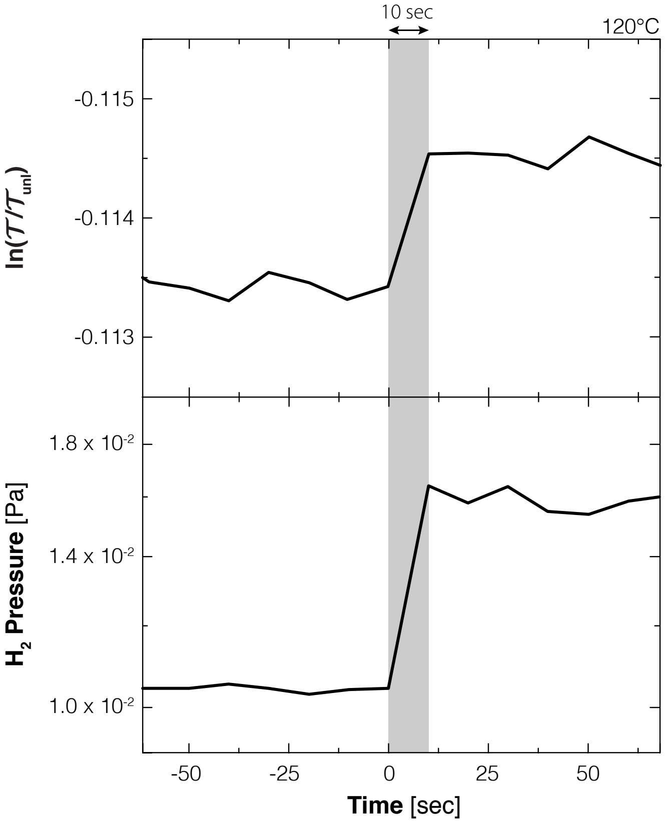

Supplementary Figure 6 | Low Pressure Kinetics. The optical response time (in seconds) of a Pd-capped 40 nm Hf thin film to a small pressure increase around 10^{-2} Pa at 120°C. The transmission $\mathcal{T}$ of the film is compared to its value at the unloaded state ($\mathcal{T}_{\mathrm{unl}}$). The gray area indicates the response time of both the pressure and the optical transmission change.

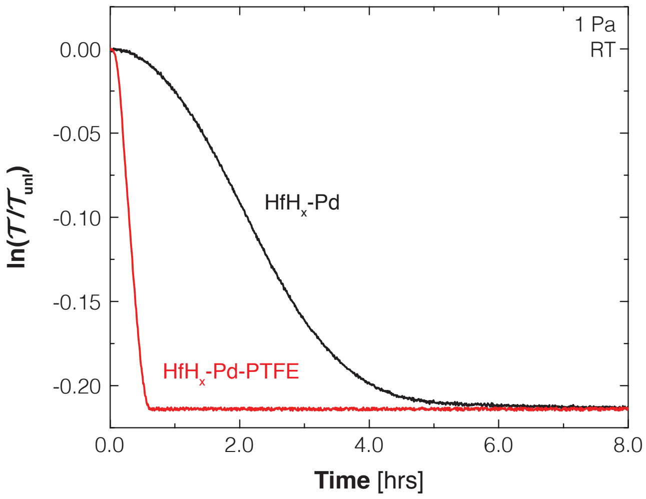

Supplementary Figure 7 | Kinetics at Room Temperature. The optical response time (in hours) of two Pd-capped 40 nm Hf thin films to a constant pressure of 1 Pa at room temperature. One of the films is capped by an additional PTFE layer (30 nm). The transmission $\mathcal{T}$ of both films is compared to their values at the unloaded state ($\mathcal{T}_{\mathrm{unl}}$). The optical response of both films is recorded simultaneously with hydrogenography and both films have the same history with respect to hydrogen/oxygen exposures.

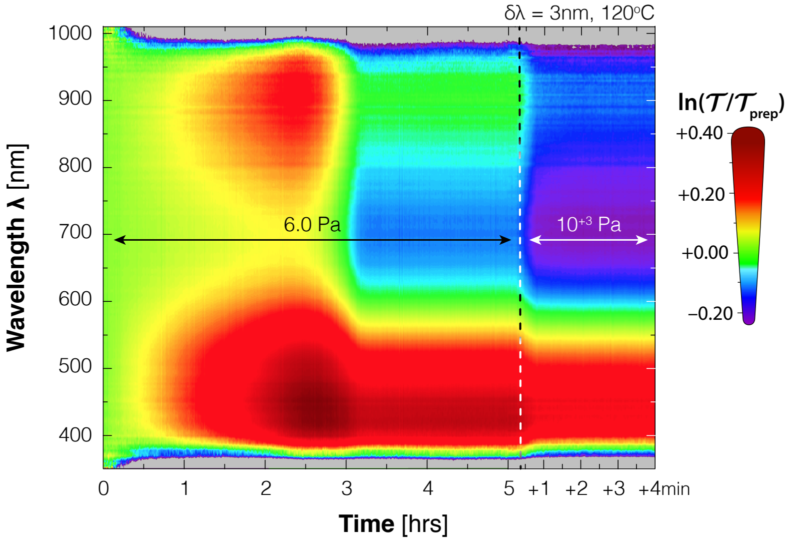

Supplementary Figure 8 | Optical Transmission Change as a function of the Wavelength. The change of the optical transmission $\mathcal{T}$ of an as-deposited Pd-capped 40 nm Hf thin film at 120°C as a function of the wavelength, exposed to an increasing hydrogen pressure P. For the first five hours, the film was exposed to a pressure of 6.0 Pa. The next few minutes it was exposed to a pressure of 10^{+3} Pa. Red indicates high transmission change with respect to the as-prepared transmission $\mathcal{T}_{\mathrm{prep}}$ of the film. Purple indicates a negative change.

Supplementary Tables

Supplementary Table 1 | Bulk Lattice Expansion. Change in the volume per formula unit and the density as a function of hydrogen content at room temperature as described by Mintz for bulk HfH_x[1]. The cell volume increases up to the fcc $\rightarrow$ fct transition. A small decrease in the volume of the unit cell is observed at this transition, after which the cell volume increases upon approaching full hydrogenation.

Compound	Structure	$V_{FU} \times 10^{-24}$ [cm^3]	ρ [g cm^{-3}]
Pure Hf (α-phase)	hcp	22.39	13.24
$HfH_{1.63}$ (δ-phase)	fcc	25.97	11.52
$HfH_{1.83}$ (δ-phase)	fcc	26.61	11.25
$HfH_{1.88}$ (ε-phase)	fct	26.33	11.38
$HfH_{1.99}$ (ε-phase)	fct	26.45	11.33

Supplementary Table 2 | X-Ray Diffraction Peak Identification. Identification of the 2θ-peak positions found in the XRD patterns shown in Supplementary Fig. 1 by means of the JCPDS-database, using the data reported for using the data reported for bulk[2]. The symbols ⬣, ■, ▬ (all HfH_x) en ■ (Pd) indicate the structures and correspond to the same symbols as in Supplementary Fig. 1. Note that for the *unloaded* state ($HfH_{1.43}$) the observed peak positions are compared to the peak position reported for bulk $HfH_{1.62}$.

	Structure	Thin Film	Bulk[2]
As Prepared (air, RT)			
- Hf (100)	⬣ hcp	37.43°	37.71°
- Hf (002)	⬣ hcp	40.96°	41.43°
- Pd (111)	■ fcc	46.84°	46.93°
Saturated (10^{+3} Pa, 120°C)			
- $Hf_{1.98}$ (111)	▬ fct	38.30°	38.40°
- Pd (111)	■ fcc	46.74°	46.93°
Unloaded (3 days in air, RT)			
- $HfH_{1.43}$ (111)	■ fcc	38.37°	38.67°
- Pd (111)	■ fcc	46.81°	46.93°
Unloaded (6 months in air, RT)			
- $HfH_{1.43}$ (111)	■ fcc	38.47°	38.67°
- Pd (111)	■ fcc	46.67°	46.93°

Supplementary Notes

Entropic Contributions

Applying the Van 't Hoff relation to the temperature dependence of the pressure-transmission-isotherms implies that ΔH does not depend on the hydrogen fraction x in HfH_x, and that the sensing range is fully due to a large entropy change upon hydrogenation. Usually, the entropy change is dominated by the entropy of hydrogen in the gas phase. Here, we investigate whether two well-known contributions to the change of the entropy - the configurational and vibrational entropy of dissolved hydrogen in the HfH_x lattice - are large enough to explain the entropy observed.

In this approach, we consider only the entropy change within the fcc-phase for $1.82 < x < 1.94$, and disregard any lattice deformation in this phase. This range corresponds to our observed data where we derive a change in entropy from -184 to -222 J (K mol $H_2)^{-1}$. In addition we will consider $x = 1.43$, where we find an extrapolated $\Delta S = -79.9$ J (K mol $H_2)^{-1}$. The definition for ΔS within the solubility range is given by Supplementary Eq. (S19b). In the limit of $\delta x \to 0$, ΔS can be simplified to the molar entropy difference between atomic hydrogen (in the metal host) and the entropy of the gas:

$$\Delta S = 2\bar{S}_{H_x} - \bar{S}^0_{H_2} \tag{S1}$$

where $\bar{S}^0_{H_2}$ is the standard molar entropy of the hydrogen gas (131 J (K mol $H_2)^{-1}$). We define the partial molar entropy of the hydride phase as the sum of the configurational and vibrational entropy which both depend on x:

$$\bar{S}_{H_x} = \bar{S}_{\text{conf}}(x) + \bar{S}_{\text{vib}}(x) \tag{S2}$$

Configuration Entropy

The configurational entropy Sconf accounts for all configurations of hydrogen in the fcc lattice and is given by:

$$\bar{S}_{\text{conf}}(x) = -R\ \ln\left[\frac{x}{r-x}\right] \tag{S3}$$

Here, r is the maximum hydrogen fraction within the system. With HfH_x being a tetrahedral system, $r = 2$. In this way we find:

$$\bar{S}_{\text{conf}}(x = 1.43) = -7.65 \text{ J (K mol H}_2)^{-1} \tag{S4a}$$

$$\bar{S}_{\text{conf}}(x = 1.80) = -18.3 \text{ J (K mol H}_2)^{-1} \tag{S4b}$$

$$\bar{S}_{\text{conf}}(x = 1.94) = -29.8 \text{ J (K mol H}_2)^{-1} \tag{S4c}$$

This result in

$$\Delta S(x = 1.43) = 2 \times (-7.61) - 131 = -\ 146 \text{ J (K mol H}_2)^{-1}$$
$$\textit{exp. -79.9 J (K mol H}_2)^{-1} \tag{S5a}$$

$$\Delta S(x = 1.80) = 2 \times (-18.3) - 131 = -\ 168 \text{ J (K mol H}_2)^{-1}$$
$$\textit{exp. -180 J (K mol H}_2)^{-1} \tag{S5b}$$

$$\Delta S(x = 1.94) = 2 \times (-29.8) - 131 = -\ 189 \text{ J (K mol H}_2)^{-1}$$
$$\textit{exp. -228 J (K mol H}_2)^{-1} \tag{S5c}$$

For none of the considered states the calculated ΔS matches the experimental values.

The expression for the configurational entropy depends on the model used. E.g., Boureau[3] presented a model taking into account the hard-core repulsion between screened protons up to second nearest neighbors:

$$\bar{S}_{\text{conf}}(x) = -R\ \ln\left[\frac{(6-x)\,x}{(6-4\ x)^2}\right] \tag{S6}$$

This leads to

$$\bar{S}_{\text{conf}}(x = 1.43) = -38.8 \text{ J (K mol H}_2)^{-1} \tag{S7a}$$

$$\bar{S}_{\text{conf}}(x = 1.80) = -13.5 \text{ J (K mol H}_2)^{-1} \tag{S7b}$$

$$\bar{S}_{\text{conf}}(x = 1.94) = -7.76 \text{ J (K mol H}_2)^{-1} \tag{S7c}$$

which shows that the contribution of the configurational entropy at $x = 1.43$ is stronger than at $x = 1.94$. This means that the entropy at $x = 1.43$ would be more negative than at $x = 1.94$:

$$\Delta S(x = 1.43) = 2 \times (-36.8) - 131 = -\,205 \text{ J (K mol H}_2)^{-1} \quad \textit{exp. -79.9 J (K mol H}_2\textit{)}^{-1} \tag{S8a}$$

$$\Delta S(x = 1.80) = 2 \times (-13.5) - 131 = -\,158 \text{ J (K mol H}_2)^{-1} \quad \textit{exp. -180 J (K mol H}_2\textit{)}^{-1} \tag{S8b}$$

$$\Delta S(x = 1.94) = 2 \times (-7.76) - 131 = -\,147 \text{ J (K mol H}_2)^{-1} \quad \textit{exp. -228 J (K mol H}_2\textit{)}^{-1} \tag{S8c}$$

This is opposite to our observations. In general, we find that the difference in entropy generated by configurational terms is too small to describe the large pressure range where there is a linear relation.

Vibrational Entropy

The vibrational entropy describes the contribution of the vibration of the hydrogen atoms within the hafnium lattice and it is given by:

$$\bar{S}_{\text{vib}}(x) = -\frac{3R}{2}\left\{\ln\left[1 - \exp\left(-\frac{\mathcal{T}}{\mathcal{T}_E(x)}\right)\right] - \frac{\mathcal{T}}{\mathcal{T}_E(x)}\frac{1}{\exp\left(-\frac{\mathcal{T}}{\mathcal{T}_E(x)}\right) - 1}\right\} \tag{S9}$$

Here, T_E is the Einstein temperature, defined as the temperature where all vibration modes are excited assuming that all modes consist of one common frequency. However, it is known that complex systems have modes consisting of multiple unique frequencies, which results in a higher temperature to excite all frequency modes. This model is known as the Debye Model. Using a rough simplification and considering only temperatures far above zero Kelvin, the Einstein temperature can be expressed in terms of the Debye temperature T_D by:

$$T_E(x) = \sqrt[3]{\frac{\pi}{6}}T_D(x) \tag{S10}$$

The Debye temperature of different hydrogen fractions within the Hf lattice is recently calculated by Wang *et al.*[4] From $x = 1.5$ to $x = 1.75$, the Debye temperature reduces from 339 K to 195 K. A linear extra/interpolation leads to $T_\text{D}(x = 1.43) = 379$ K, $T_\text{D}(x = 1.80) = 166$ K and $T_\text{D}(x = 1.94) = 86$ K, or equivalent to $T_\text{E}(x = 1.43) = 305$ K, $T_\text{E}(x = 1.80) = 134$ K and $T_\text{E}(x = 1.94) = 69$ K. Inserting these temperatures in Supplementary Eq. S9, we find that:

$$\bar{S}_{\text{vib}}(x = 1.43) = -14.4 \text{ J (K mol H}_2)^{-1} \tag{S11a}$$

$$\bar{S}_{\text{vib}}(x = 1.80) = -18.0 \text{ J (K mol H}_2)^{-1} \tag{S11b}$$

$$\bar{S}_{\text{vib}}(x = 1.94) = -53.0 \text{ J (K mol H}_2)^{-1} \tag{S11c}$$

which results in:

$$\Delta S(x = 1.43) = 2 \times (-14.4) - 131 = -\,205 \text{ J (K mol H}_2)^{-1} \quad \textit{exp. -160 J (K mol H}_2\textit{)}^{-1} \tag{S12a}$$

$$\Delta S(x = 1.80) = 2 \times (-18.0) - 131 = -\,167 \text{ J (K mol H}_2)^{-1} \quad \textit{exp. -180 J (K mol H}_2\textit{)}^{-1} \tag{S12b}$$

$$\Delta S(x = 1.94) = 2 \times (-53.0) - 131 = -\,237 \text{ J (K mol H}_2)^{-1} \quad \textit{exp. -228 J (K mol H}_2\textit{)}^{-1} \tag{S12c}$$

Again, the calculated values are not in agreement with the experimental found values. Even if we combine the configurational and vibrational contributions we cannot describe the pressure range found experimentally:

$$\Delta S(x = 1.43) = 2 \times (-7.61 - 14.4) - 131 = -\ 175\ \text{J (K mol H}_2)^{-1}$$
$$\textit{exp. -160 J (K mol H}_2)^{-1} \quad \text{(S13a)}$$
$$\Delta S(x = 1.80) = 2 \times (-18.3 - 18.0) - 131 = -\ 225\ \text{J (K mol H}_2)^{-1}$$
$$\textit{exp. -180 J (K mol H}_2)^{-1} \quad \text{(S13b)}$$
$$\Delta S(x = 1.94) = 2 \times (-29.8 - 53.0) - 131 = -\ 295\ \text{J (K mol H}_2)^{-1}$$
$$\textit{exp. -228 J (K mol H}_2)^{-1}. \quad \text{(S13c)}$$

as well for using Boureau's model for the configurational entropy:

$$\Delta S(x = 1.43) = 2 \times (-36.8 - 14.4) - 131 = -\ 233\ \text{J (K mol H}_2)^{-1}$$
$$\textit{exp. -160 J (K mol H}_2)^{-1} \quad \text{(S14a)}$$
$$\Delta S(x = 1.80) = 2 \times (-13.5 - 18.0) - 131 = -\ 194\ \text{J (K mol H}_2)^{-1}$$
$$\textit{exp. -180 J (K mol H}_2)^{-1} \quad \text{(S14b)}$$
$$\Delta S(x = 1.94) = 2 \times (-7.8 - 53.0) - 131 = -\ 253\ \text{J (K mol H}_2)^{-1}$$
$$\textit{exp. -228 J (K mol H}_2)^{-1}. \quad \text{(S14c)}$$

Supplementary Methods

DFT Calculations

The Lambert-Beer law relates the transmission $\mathcal{T}$ of a film to its thickness d and the absorption coefficient α:

$$\ln\left(\frac{\mathcal{T}}{\mathcal{T}_0}\right) = -\alpha(x)\, d(x) \tag{S15}$$

Here, $\mathcal{T}_0$ takes into account the transmission losses of the system, including the reflection at all interfaces. The absorption coefficient α is given by

$$\alpha(x) = \frac{\sqrt{2}\omega}{c}\left(\sqrt{\varepsilon_{\mathrm{r}}^2\,(x) + \varepsilon_{\mathrm{i}}^2\,(x)} - \varepsilon_{\mathrm{r}}\,(x)\right) \tag{S16}$$

Here, c is the speed of light, ω the angular frequency, and ε_{r} and ε_{i} are, respectively, the real and imaginary part of the dielectric function:

$$\varepsilon\,(x) = \varepsilon_{\mathrm{r}}\,(x) + i\;\varepsilon_{\mathrm{i}}\,(x) \tag{S17}$$

Per definition, the dielectric constants are not only a function of x but also of ω. Supplementary Fig. 3a shows the wavelength dependence of ε_{i} calculated for HfH_2 with a (theoretical) cubic (fcc) and a tetragonal (fct) structure. The calculations show that ε_{i} is similar for both structures. This implies that the fcc-fct phase transition should not result in a significant optical effect. Calculating the dielectric function for fcc $HfH_{1.5}$ we observe a clear change in ε_{i}, especially for energies below 2 eV.

When we plot the density of states and compare for two hydrogen fractions ($x = 1.5$ and $x = 2$) we observe that the Fermi level decreases by 2.1 eV, implying that the dielectric function depends indeed on the hydrogen fraction (see Supplementary Fig. 3b). At the Fermi level, the DOS does not dramatically change. This implies that we do not expect large changes in the electronic H-H interaction as a function of x.

Deriving the apparent Enthalpy and Entropy using the Van 't Hoff Construction

The Van 't Hoff relation describes the temperature dependence of the equilibrium hydrogen pressure P in metal hydrides:

$$\ln\left(\frac{P}{P_0}\right) = -\frac{\Delta H}{R\,T} - \frac{\Delta S}{R} \tag{S18}$$

Here, ΔH and ΔS are the enthalpy and entropy of hydride formation, both expressed per mol H_2. Within the fcc solubility range ΔH is simply defined as the heat of dissolving hydrogen in the hydride states and ΔS is the difference between the partial molar entropy of the hydride states with respect to standard molar entropy of the hydrogen gas:

$$\Delta H = 2\frac{\bar{H}_{x+\delta x} - \bar{H}_x}{\delta x} - \bar{H}^0_{H_2} \tag{S19a}$$

$$\Delta S = 2\frac{\bar{S}_{x+\delta x} - \bar{S}_x}{\delta x} - \bar{S}^0_{H_2} \tag{S19b}$$

Here, $\bar{S}^0_{H_2}$ is the standard molar entropy of the hydrogen gas (131 J (K mol H_2)$^{-1}$), $\bar{H}^0_{H_2}$ the standard molar entropy of the hydrogen gas, and $\bar{H}_x$ and $\bar{S}_x$, respectively, the molar enthalpy and entropy of the dissolved hydrogen at fraction x. The Pressure-Transmission-Isotherms (P$\mathcal{T}$Is) obtained at the three temperatures in Fig. 5 allow us to construct a Van 't Hoff plot (Supplementary Fig. 4a), which relates the log hydrogen pressure to the inverse temperature for a specific hydrogen fraction. Here, we use the fact that each transmission value represents a certain fraction x, since the relation between x and the optical transmission $\mathcal{T}$ will not change in the small

temperature range involved. Selecting from different compositions we plot in Supplementary Fig. 4a the Van 't Hoff lines for four different compositions. The lines run parallel since the three P$\mathcal{T}$Is of Fig. 5 can be normalized to the 120°C data by a vertical simple shift (Supplementary Fig. 2). This implies a constant enthalpy for the whole measured compositional range.

From the slope of the Van 't Hoff plots (Supplementary Fig. 4a) we derive the enthalpy of hydrogenation. In Supplementary Fig. 4b these values are plotted for all available data sets, resulting in an average enthalpy of formation equal to $\Delta H = -118$ kJ (mol H_2)$^{-1}$, which is close to the enthalpy of dissolution $\Delta_f H = -112$ kJ (mol H_2)$^{-1}$ reported for bulk samples at high temperatures[1].

Similarly, from the extrapolation to infinite temperature we derive a value for the entropy of hydrogenation. Clearly, it follows that the increase of the hydrogen equilibrium pressure as a function of $\ln(\mathcal{T}/\mathcal{T}_{\rm unl})$ is fully determined by the increase in entropy. Since the neutron data showed a linear relation between x and $\ln(P/P_0)$, the entropy ΔS appears to be a linear function of the hydrogen fraction x and ranges from -180 J (K mol H_2)$^{-1}$ at $x = 1.8$, to -250 J (K mol H_2) $^{-1}$ at $x = 1.98$ (see Supplementary Fig. 4b). The latter values appear to be unphysically large. Note, that the value we obtain on extrapolation to $x = 1.43$ is close to that obtained by Mintz (-79.9 J (K mol H_2)$^{-1}$ versus -82.8 J (K mol H_2)$^{-1}$)[1].

Supplementary References

[1] Mintz, M. H., Hafnium-hydrogen. *Sol. State Phenom.* **49-50**, 331-356 (1996).

[2] JCPDS-lnternational Centre for Diffraction Data (1998).

[3] Boureau, G. The configurational entropy of hydrogen in body centered metals. *J. Phys. Chem. Solids* **45**, 973-974 (1984).

[4] Wang, H. & Konashi, K. Investigation on electronic, mechanical and thermal properties of Hf-H system. *J. Nuc. Mat.* **443**, 99-106 (2013).
